# Diet Quality and Advanced Periodontitis in Relation to Serum CRP Levels: An 11‐Year Follow‐Up Study

**DOI:** 10.1002/fsn3.71762

**Published:** 2026-04-13

**Authors:** Sanna Syrjäläinen, Satu Männistö, Eija Könönen, Anna Liisa Suominen, Ulvi Kahraman Gürsoy

**Affiliations:** ^1^ Periodontology, Institute of Dentistry University of Turku Turku Finland; ^2^ Department of Public Health Finnish Institute for Health and Welfare Helsinki Finland; ^3^ Institute of Dentistry University of Eastern Finland Kuopio Finland; ^4^ Oral and Maxillofacial Teaching Unit Kuopio University Hospital Kuopio Finland

**Keywords:** Baltic Sea diet score, C‐reactive protein, diet, dietary inflammatory index, periodontitis

## Abstract

This study aimed to evaluate the combined association of diet and advanced periodontitis with serum high‐sensitivity C‐reactive protein (hs‐CRP) levels at baseline and after 11 years. Study participants (*n* = 3058 at baseline and *n* = 3007 at follow‐up) were drawn from the Finnish population‐based Health 2000 and 2011 surveys. Baltic Sea Diet Score (BSDS), Dietary Inflammatory Index, and periodontal status were used as exposure variables, with hs‐CRP serum levels as the outcome. Baseline CRP levels in participants, categorized into four groups according to exposure variables, were compared with the Kruskal–Wallis test. The association of dietary indices and advanced periodontitis with serum CRP levels after 11 years of follow‐up was analyzed with linear regression. Participants with advanced periodontitis (at least one tooth with ≥ 6 mm pocket depth) had higher hs‐CRP levels in serum at baseline, in comparison to controls. The concurrent exposure to low BSDS or proinflammatory diet and advanced periodontitis was not associated with further increase in serum CRP levels. At an 11‐year follow‐up, unadjusted regression models demonstrated associations between elevated CRP levels and advanced periodontitis (*β* = 0.19; 95% CI 0.08–0.31 and *β* = 0.20; 95% CI 0.09–0.31) or advanced periodontitis combined with low BSDS (*β* = 0.23; 95% CI 0.09–0.37) or proinflammatory diet (*β* = 0.18; 95% CI 0.02–0.34). The statistically significant differences disappeared after controlling for confounding factors. In conclusion, combined exposure to low BSDS or a proinflammatory diet and advanced periodontitis was not significantly associated with elevated hs‐CRP levels compared to single risk factor exposure.

## Graphical abstract

This study examined whether concurrent exposure to two independent risk factors of systemic inflammation, poor diet quality and advanced periodontitis, is associated with elevated serum hs‐CRP levels at baseline and after 11 years of follow‐up. Participants with advanced periodontitis had higher hs‐CRP levels in serum at baseline, in comparison to controls. The concurrent exposure to poor diet and advanced periodontitis was not associated with further increase in serum CRP levels. After 11‐year follow‐up, unadjusted regression models demonstrated associations between elevated CRP levels and advanced periodontitis or advanced periodontitis combined with low Baltic Sea Diet Score (BSDS), but the statistically significant differences disappeared after controlling for confounding factors.

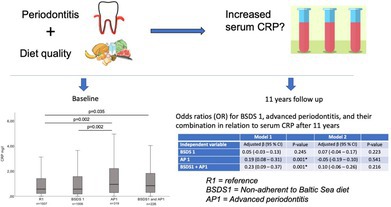

## 1 Introduction

C‐reactive protein (CRP), an acute‐phase protein, is produced by hepatocytes in response to inflammatory stimulus (Black et al. 2004). Throughout their lifespan, individuals are exposed to various modifiable lifestyle risk factors, such as smoking, physical inactivity, stress, obesity, and inadequate sleep, which are related to increased circulating CRP in serum. Driven by chronic systemic inflammation, they are causally linked to the development of many chronic diseases, among them type 2 diabetes and cardiovascular diseases (Furman et al. 2019).

Periodontitis is a chronic disease affecting tooth‐supporting tissues (connective tissue, periodontal ligament, and alveolar bone) and, if left untreated, it can cause substantial tissue destruction and tooth loss (Könönen et al. 2019). It is initiated by the interplay between dysbiotic biofilm at the gingival margin and host response where uncontrolled inflammatory reaction is accompanied by excessive production of inflammatory mediators (Jaedicke et al. 2015). Further, these mediators can enter the bloodstream, causing a systemic inflammatory response (Zhu et al. 2025). The relationship between immune response and periodontitis is bidirectional, and the complexity of the relation to systemic diseases arises from the possibility that many systemic conditions increase the risk of developing periodontitis (Hajishengallis 2022; Hasan et al. 2025).

Unfavorable nutrition, commonly described as high intake of saturated fatty acids and/or low intake of fruits and vegetables, polyphenols, and other antioxidants, is another significant risk factor for low‐grade inflammation (Calder et al. 2009; Minihane et al. 2015). Diet quality has been shown to strongly affect the risk for immune‐mediated systemic diseases (Billingsley et al. 2024; Forouhi 2023; Seth et al. 2025). Thus, diet could affect the risk for periodontitis progression as well. The overall diet quality can be assessed using culture‐specific indices, such as the Baltic Sea Diet Score (BSDS) in Nordic countries (Kanerva, Kaartinen, et al. 2014). Another approach is to assess the diet's inflammatory potential by the Dietary Inflammatory Index (DII), which identifies the anti‐ and proinflammatory components of the diet (Shivappa et al. 2014). In a Finnish study of 4579 adults, elevated CRP levels were observed in participants who were not adherent to the Baltic Sea diet assessed by BSDS (Kanerva, Loo, et al. 2014). Likewise, several large‐scale studies have shown DII to be associated with increased inflammatory markers in serum, including CRP, interleukin (IL)‐6, and tumor necrosis factor (TNF)‐α (Doustmohammadian et al. 2024; Lécuyer et al. 2023; Millar et al. 2022). The association between overall diet quality (here: adherence to the Mediterranean diet or anti‐inflammatory diet) and inflammatory biomarkers was reported in a systematic review of 69 observational studies (Hart et al. 2021a). Furthermore, studies with both cross‐sectional and long‐term follow‐up designs indicated that periodontitis, and especially its aggressive forms (Salzberg et al. 2006), is strongly associated with elevated serum CRP levels (Haro et al. 2012; Machado et al. 2021; Molinsky et al. 2022).

In a cohort study of 1014 patients with coronary artery disease, a significant increase in serum CRP levels was observed in those having at least two modifiable risk factors of systemic inflammation (smoking, lack of physical activity, overweight, or poor adherence to the Mediterranean diet) in comparison to those having only one risk factor (Blaum et al. 2021). Moreover, no difference in serum CRP concentration was recognized between patients without modifiable risk factors and those with only one risk factor. Given that low‐grade systemic inflammation may result from diverse etiological factors, a multifaceted approach accounting for various lifestyle exposures is essential to reveal the real association of exposure with elevated CRP levels and the subsequent systemic disease burden.

In the present study, we hypothesized that inflammatory burden from advanced periodontitis, combined with non‐adherence to the Baltic Sea diet or adherence to the proinflammatory diet increases their risk for elevated high‐sensitivity (hs) CRP in serum. Our objective was to examine whether concurrent exposure to two independent risk factors of systemic inflammation, namely the lower BSDS or higher DII and advanced periodontitis, is associated with elevated serum hs‐CRP levels at baseline and after 11 years of follow‐up.

## 2 Materials and Methods

### 2.1 Study Sample

**FIGURE 1:**
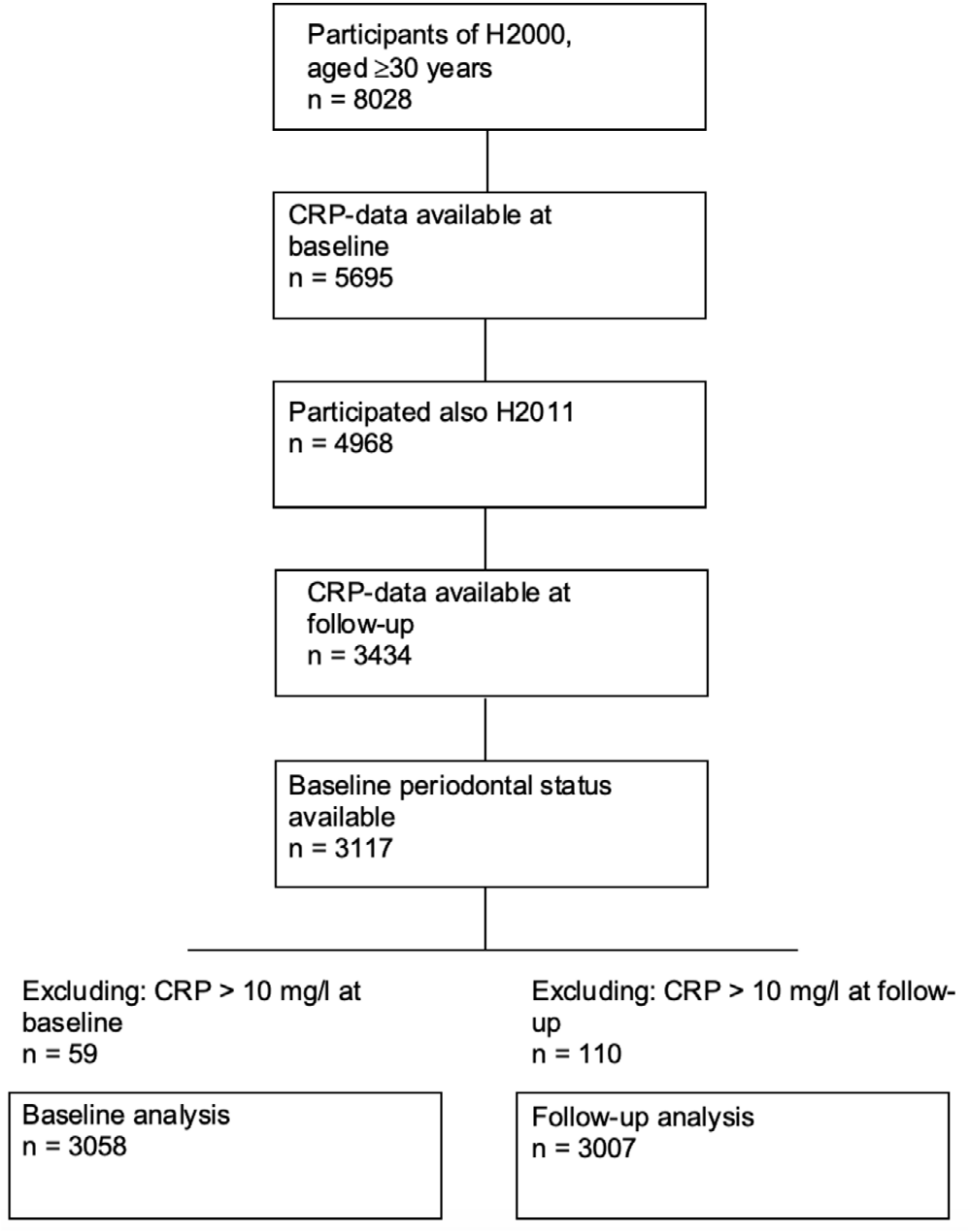
Flowchart for the selection of study participants.

This cohort study is based on the Finnish population‐based Health 2000 (H2000) and Health 2011 (H2011) surveys (Heistaro 2008; Lundqvist and Mäki‐Opas 2016). Participants were selected using a two‐stage stratified sampling from the Finnish Population Registry in 2000–2001, resulting in a sample size of 8028 aged 30 years or over. From the whole sample, 6986 (87%) took part in the interview and 6770 (84%) in the health examination either at the study site or home. All those attending the main H2000 survey, who were alive, were living in Finland on 6 July 2011, had available contact details, and had not refused further participation, were invited to join the H2011 follow‐up during 2011 and 2012 (*n* = 8135). Of them, 5903 (74%) accepted the invitation. The current study includes participants who were 30 years or older at baseline, as they form the main cohort of H2000. Due to the integral role of periodontitis in the present study, we included only those who had periodontal status measured at the baseline. In addition, we included only participants who had both baseline and follow‐up hs‐CRP levels measured (Figure 1). Furthermore, we excluded those with hs‐CRP > 10 mg/L reflecting an acute infection (Ridker 2016), resulting in 3058 participants for baseline analyses and 3007 participants for follow‐up analyses.

Both H2000 and H2011 surveys included health examinations in the field, where the health measurements were made, and fasting blood samples were collected. In H2000, home‐visit interviews were made, whereas questionnaires were filled during health examinations in H2011 (Heistaro 2008; Lundqvist and Mäki‐Opas 2016).

Both surveys were approved by the Ethical Committee of the Hospital District of Helsinki and Uusimaa (Health 2000, 15.7.2004 reference 407/E3/2000; and Health 2011, 22.3.2011 reference 45/13/03/00/11), and a written informed consent was obtained from each participant. This study has been reported in accordance with the STROBE guidelines.

### 2.2 Baltic Sea Diet Score and Dietary Inflammatory Index

The participants completed a validated semi‐quantitative food frequency questionnaire (FFQ) at home to assess their usual food consumption over the last 12 months (Kaartinen et al. 2012; Männistö et al. 1996). The FFQ covered 131 food items or mixed dishes with nine frequency categories ranging from “never or seldom” to “at least six times a day”. The portion size was fixed for each food item or mixed dish (e.g., slice and glass). The dietary data were converted into average daily food consumption and nutrient intake using the National Food Composition Database, Fineli, and the Finessi software of the Finnish Institute for Health and Welfare (Reinivuo et al. 2010).

Two indices were used to assess the overall diet quality. The BSDS, evaluating the adherence to the Baltic Sea diet, included nine dietary components typically grown or eaten in Nordic countries (Kanerva, Kaartinen, et al. 2014). In the present study, we used the modified BSDS, which corresponds more closely to Nordic dietary recommendations (Männistö et al. 2021). In the modified BSDS, the total fat intake was excluded from the index as the quality of fat is more important from the health perspective. The fat quality is included in the index as a ratio of polyunsaturated fatty acids to saturated fatty acids and trans‐fatty acids. Intake of each component, except alcohol, was scored according to sex‐specific consumption quartiles: the lowest quartile was coded as 0, the second one as 1, the third one as 2, and the highest one as 3. For meat products, the scoring was reversed. Alcohol consumption was assigned based on the Finnish nutrition recommendations published in 2014 (National Nutrition Council 2021), where men consuming ≤ 20 g and women consuming ≤ 10 g of ethanol per day received 1 point, while others received 0 points. The final BSDS ranged from 0 to 22, with higher scores representing a greater adherence to the Baltic Sea diet.

The calculation of DII was conducted according to Shivappa et al. (2014) with a few modifications we have described previously (Syrjäläinen et al. 2024). In the present study, the DII calculation was based on 29 of the original 45 food components included in the index. These food components were alcohol, beta‐carotene, carbohydrate, cholesterol, energy, fiber, folic acid, green/black tea, iron, isoflavones, magnesium, monounsaturated fatty acids, niacin, n‐3 fatty acids, n‐6 fatty acids, protein, polyunsaturated fatty acids, riboflavin, saturated fatty acids, selenium, thiamin, trans fats, vitamin A, vitamin B6, vitamin B12, vitamin C, vitamin D, vitamin E, and zinc. The missing food components were mainly spices (e.g., garlic, ginger, or saffron) or some classes of flavonoids (e.g., flavones or flavonols) as their amount were not able to be measured by FFQ.

### 2.3 Periodontal Status

Periodontal status was assessed as a part of the oral health examination of H2000 by five field teams, each including one dentist who performed the clinical examinations (Syrjäläinen et al. 2024). The clinical procedure has been described elsewhere (Syrjäläinen et al. 2024). Briefly, probing pocket depth (PPD) was measured from four sites of each tooth, and the deepest PPD of any tooth was recorded to diagnose periodontitis as follows: non‐periodontitis (PPD ≤ 3 mm at all teeth), mild to moderate periodontitis (PPD of 4–5 mm at any tooth), or advanced periodontitis (PPD ≥ 6 mm at any tooth). The quality assurance was ensured by a reference dentist, who took parallel measurements of pocket depths during several visits to each field team, the agreement being 77% (κappa‐value 0.41) between the reference dentist and the field dentist. Furthermore, the repeated measurements for randomly selected participants showed a κappa‐value of 0.83.

### 2.4 Categorization of Study Participants

To classify participants according to their risk factors for elevated serum CRP, participants were categorized into four groups according to the non‐adherence to BSDS or adherence to proinflammatory diet and advanced periodontitis. Before categorization, the participants were divided into tertiles according to their BSDS or DII scores, where BSDS tertile 1 represents those with the least adherence to the Baltic Sea diet. In contrast, DII tertile 3 represents those with the most proinflammatory diet. In the analyses evaluating the association of the Baltic Sea diet in combination with advanced periodontitis, the participants were grouped as follows:

- *The reference group (R1)*: participants who adhered to the Baltic Sea diet (BSDS tertiles 2 or 3) and were not diagnosed with advanced periodontitis (non‐periodontitis or moderate periodontitis).
- *Non‐adherence to the Baltic Sea diet group (BSDS 1)*: participants who were at BSDS tertile 1 and not diagnosed with advanced periodontitis.
- *Advanced periodontitis groups (AP1)*: participants who were diagnosed with advanced periodontitis and adhered to the Baltic Sea diet (BSDS tertile 2 or 3).
- *Non‐adherence to the Baltic Sea diet and advanced periodontitis group (BSDS 1 and AP1)*: participants who were at BSDS tertile 1 and diagnosed with advanced periodontitis.

In the analyses evaluating the association of proinflammatory diet combined with advanced periodontitis, the participants were grouped as follows:

- *The reference group (R2)*: participants who adhered to an anti‐inflammatory diet (DII tertiles 1 or 2) and were not diagnosed with advanced periodontitis (non‐periodontitis or moderate periodontitis).
- *Proinflammatory diet group (DII3)*: participants who adhered to a proinflammatory diet (DII tertile 3) and were not diagnosed with advanced periodontitis.
- *Advanced periodontitis group (AP2)*: participants who were diagnosed with advanced periodontitis and adhered to an anti‐inflammatory diet (DII tertile 1 or 2).
- *Proinflammatory diet and advanced periodontitis group (DII3 and AP2)*: participants who adhered to a proinflammatory diet (DII tertile 3) and had advanced periodontitis.

### 2.5 Serum C‐Reactive Protein

In the H2000 and H2011 surveys, serum samples were collected after 4 h of fasting. The samples were centrifuged at examination sites, frozen to −20°C, and transferred within 2 weeks to their final storage location where they were stored at −70°C until analyses. In H2000, hs‐CRP was determined using an ultrasensitive immunoturbidometric test (Orion Diagnostica, Espoo, Finland) with Optima analyzer (Thermo Electron Corporation, Vantaa, Finland), and in Health 2011 with an immunoturbidometric test (CRP Vario) with Abbott c8000 analyzer (Abbott Laboratories, Abbott Park, IL, USA). The lowest limit of detection (LLOD) was 0.2 mg/L, and all values below LLOD were substituted with 0.1 mg/L. The cut‐off of 2 mg/L was used to represent systemic inflammation, as this threshold has been found to be associated with a wide variety of chronic diseases and deaths (Drozd et al. 2022).

### 2.6 Covariates

Age, smoking, physical activity, education, and medication of lipid‐modifying agents were recorded in interviews. Smoking was categorized as either current smokers or non‐smokers. Leisure time physical activity was categorized into two groups: those with no physical activity or light activity (e.g., walking or cycling) for at least 4 h per week, and those with moderate physical activity for at least 3 h per week or vigorous exercise (e.g., competitive sport). Education was categorized into two groups: basic education and vocational or higher education. For body mass index (BMI) calculations, height and weight were measured in the health examination. If these measurements were unavailable, self‐reported height and weight were used. Hemoglobin A1C (HbA1c) concentration was determined with an immunoturbidimetric method (Hemoglobin A1c assay; Abbott Laboratories). Energy intake was added to analyses as a confounding factor to adjust the models with energy intake.

### 2.7 Statistical Analyses

All statistical analyses were performed with IBM SPSS Statistic 28.0 (https://www.ibm.com/products/spss‐statistics, RRID:SCR_019096). Normality for continuous variables was assessed visually using histograms. Kruskal–Wallis test and Chi square test were used in descriptive statistics to analyze the basic characteristics of participants across BSDS or DII tertiles. Bonferroni correction was applied to post hoc analysis in Chi square test. To examine the cumulative effect of the diet and periodontitis on hs‐CRP in serum, the serum hs‐CRP levels between groups were compared using Kruskal–Wallis test. Linear regression model was conducted to analyze the change in hs‐CRP levels during 11 years by DII, BSDS, and periodontitis. In addition, linear regression analysis was used to determine the association between the dietary pattern, advanced periodontitis, or combination of these two and hs‐CRP levels. For linear regression, hs‐CRP levels were log transformed due to its right skewed distribution. To examine whether categorizing diet into tertiles affects the results, a linear regression analysis was also conducted to assess the association between DII and BSDS and hs‐CRP within different periodontitis severity groups, using diet as a continuous variable. These results are presented in Tables S1 and S2.

## 3 Results

### 3.1 Baseline Analysis

Of the study participants, 1184 (38%) were non‐periodontitis, 1329 (44%) had moderate periodontitis, and 545 (18%) had advanced periodontitis. The characteristics of participants by BSDS or DII tertiles are presented in Tables 1a and 1b. In relation to participants with BSDS tertile 3 or DII tertile 1 (representing the adherence to Baltic Sea diet or the most anti‐inflammatory diet), a higher proportion of those with BSDS tertile 1 or DII tertile 3 were older or less likely to be current smokers. BMI was slightly higher among participants in the BSDS tertile 3 than tertile 1. Those having a proinflammatory diet were more often men and more likely to have basic education than those with an anti‐inflammatory diet. No differences were observed in hs‐CRP levels or in the prevalence of AP across BSDS or DII tertiles.

**TABLE 1a:** Baseline characteristic of the study population according to BSDS tertiles.

	BSDS		
	Least adherent to the Baltic Sea diet	Most adherent to the Baltic Sea diet	
	Tertile 1	Tertile 2	Tertile 3
*N*	1232	921	905
BSDS scores	1–10	11–13	14–22
Age (years), mean (SD)†	44.7 (10.2)^a^	47.4 (10.8)^b^	51.4 (11.3)^c^
Men, *n* (%)‡	542 (44.0)	423 (45.9)	423 (46.7)
Basic education, *n* (%)‡	307 (25.0)	231 (25.1)	246 (27.3)
Current smokers, *n* (%)‡	402 (32.8)^a^	218 (23.7)^b^	147 (16.3)^c^
BMI (kg/m^2^), mean (SD)†	26.2 (4.5)^a^	26.4 (4.1)^a,b^	26.7 (4.3)^b^
Advanced periodontitis, *n* (%)‡	226 (18.3)	161 (17.5)	158 (17.5)
Serum CRP (mg/L), mean (SD)†	1.3 (1.8)	1.3 (1.8)	1.2 (1.6)

*Note:* † = Kruskal–Wallis test, ‡ = Chi square test. Statistically significant differences (*p*‐value < 0.05) between the tertiles are indicated with a, b, c. Advanced periodontitis = at least one pocket depth ≥ 6 mm.

Abbreviations: BMI = body mass index, BSDS = Baltic Sea Diet Score, CRP = C‐reactive protein, SD = standard deviation.

**TABLE 1b:** Basic characteristics of the study population according to DII tertiles.

	DII		
	Most anti‐inflammatory diet	Most proinflammatory diet	
	Tertile 1	Tertile 2	Tertile 3
*N*	1030	1068	960
DII range	−6.4 to −3.8	−3.8 to −1.7	−1.7 to 4.4
Age (years), mean (SD)†	48.9 (10.0)^a^	47.0 (10.9)^b^	46.5 (11.2)^b^
Men, *n* (%)‡	435 (42.2)^a^	494 (46.3)^a,b^	459 (47.8)^b^
Basic education, *n* (%)‡	243 (23.8)^a^	264 (24.8)^a,b^	277 (28.9)^b^
Current smokers, *n* (%)‡	212 (20.7)^a^	245 (23.0)^a^	310 (32.3)^b^
BMI (kg/m^2^), mean (SD)†	26.5 (4.2)	26.5 (4.4)	26.3 (4.3)
Advanced periodontitis, *n* (%)‡	184 (17.9)	176 (16.5)	185 (19.3)
Serum CRP (mg/L), mean (SD)†	1.2 (1.7)	1.2 (1.7)	1.3 (1.8)

*Note:* † = Kruskal–Wallis test, ‡ = Chi square test. Statistically significant differences (*p*‐value < 0.05) between the tertiles are indicated with a, b, c. Advanced periodontitis = at least one pocket depth ≥ 6 mm.

Abbreviations: BMI = body mass index, CRP = C‐reactive protein, DII = Dietary Inflammatory Index, SD = standard deviation.

**FIGURE 2:**
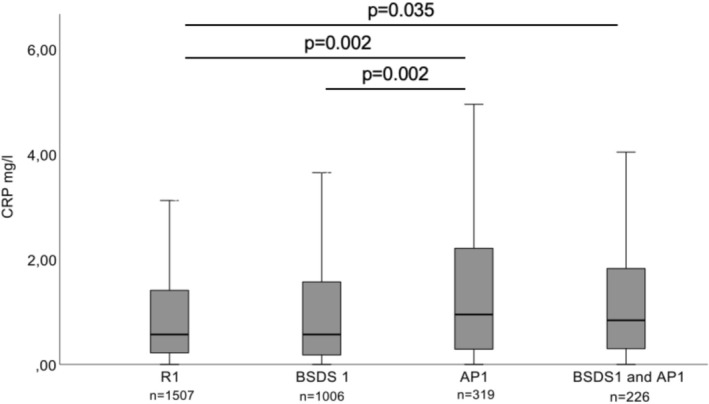
Serum CRP levels (mg/L) at baseline in participants categorized into R1, BSDS1, AP1, or BSDS 1 and AP1 groups. R1 = reference group (BSDS tertiles 2 or 3 and not diagnosed with advanced periodontitis). BSDS 1 = Non‐adherence to the Baltic Sea diet group (BSDS tertile 1). AP 1 = advanced periodontitis group (at least one pocket depth ≥ 6 mm). BSDS = Baltic Sea Diet Score. *p*‐Values from Kruskal–Wallis test. Unadjusted models. Other *p*‐values than those depicted in the figure were non‐significant (*p* > 0.05).

**FIGURE 3:**
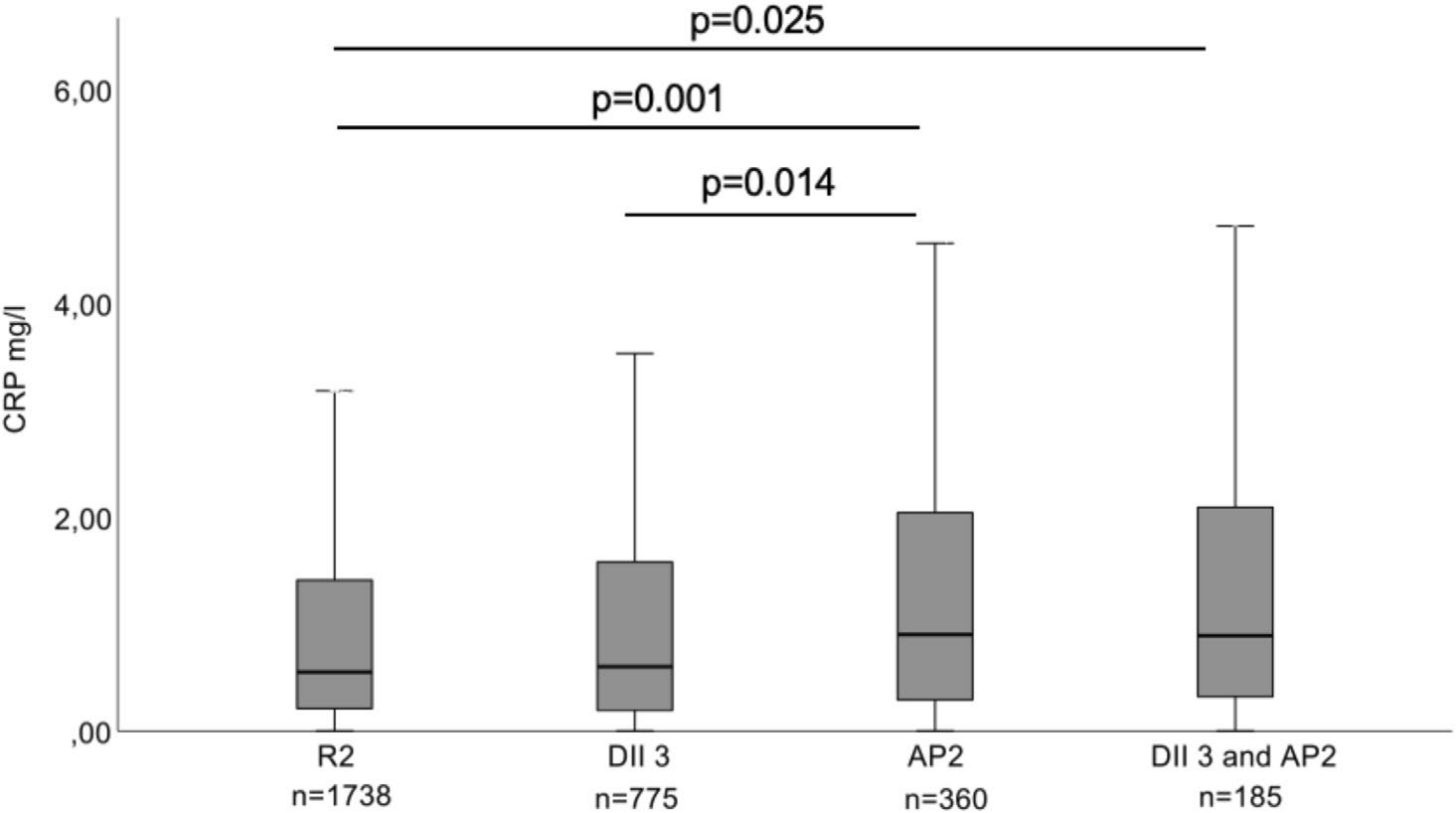
Serum CRP levels (mg/L) at baseline in participants categorized into R1, DII3, AP2, or DII3 and AP2 groups. R2 = reference group (DII tertiles 1 or 2 and not diagnosed with advanced periodontitis). DII 3 = proinflammatory diet group (DII tertile 3). AP 2 = advanced periodontitis group (at least one pocket depth ≥ 6 mm). DII = dietary Inflammatory index. *p*‐Values from Kruskal–Wallis test. Unadjusted models. Other *p*‐values than those depicted in the figure were non‐significant (*p* > 0.05).

The AP1 and AP2 groups had statistically significantly higher hs‐CRP levels compared to R1 and R2 groups in both dietary analyses (*p* = 0.002 and *p* = 0.001 respectively, Figures 2 and 3). They also had higher CRP levels than the BSDS 1 group (*p* = 0.002) or DII3 group (*p* = 0.014). When participants had two risk factors (BSDS1 and AP1 group, and DII3 and AP2 group), their CRP levels were significantly higher than in R1 and R2 groups (*p* = 0.035 and *p* = 0.025). BSDS1 and AP1 group, or DII3 and AP2 group, did not differ from those having only one risk factor (BSDS1, DII 3, AP1, and AP2 groups).

### 3.2 Eleven Years of Follow‐Up

In the 11‐year follow‐up analysis, 2477 (82%) participants had non‐ or mild to moderate periodontitis at baseline, while 530 (18%) had advanced periodontitis. The mean change in hs‐CRP levels during the follow‐up was 1.14 mg/L (SD 6.08); the levels being increased in 73.5% (mean 2.08 mg/L, SD 6.77) and decreased in 26.5% (mean −1.46 mg/L, SD 1.80) of the participants. Baseline DII, BSDS, or periodontal status did not affect the CRP change during the follow‐up (*β* = −0.180, *p*‐value = 0.38 for DII; *β* = −0.003, *p*‐value = 0.91 for BSDS; and *β* = 0.002, *p*‐value = 0.98 for periodontitis; Adjusted *R* ^2^ = −0.001, *p*‐value = 0.674).

After 11 years of follow‐up, advanced periodontitis at baseline either alone or in combination with BSDS 1 or DII 3 was slightly associated with serum CRP (AP1: *β* = 0.19, *p*‐value < 0.001; AP1 + BSDS1: *β* = 0.23, *p*‐value = 0.001; AP2: *β* = 0.20, *p*‐value < 0.001; AP2 + DII3 *β* = 0.18, *p*‐value = 0.024; Tables 2 and 3). Neither BSDS 1 nor DII 3 at baseline were associated with CRP after 11 years. Covariates‐adjusted linear regression analysis models (Models 2, adjusted for baseline age, BMI, smoking status, physical activity, education level, CRP, HbA1c, medication of lipid‐modifying agents, and energy intake) indicated non‐significant associations. In the Models 2, the main determinants of elevated hs‐CRP levels after 11 years were baseline BMI and CRP levels (data not shown). When linear regression analysis was performed using DII and BSDS as continuous variables, neither DII nor BSDS was associated with hs‐CRP within the different periodontitis severity categories (Supporting material Tables S1 and S2).

**TABLE 2:** Associations of BSDS 1, advanced periodontitis, or combination of both with serum CRP levels after 11 years of follow up.

Independent variable	Model 1		Model 2	
	Adjusted *β* (95% CI)	*p*	Adjusted *β* (95% CI)	*p*
BSDS 1	0.05 (−0.03 to 0.13)	0.245	0.07 (−0.04 to 0.17)	0.223
AP 1	0.19 (0.08 to 0.31)	0.001*	−0.05 (−0.19 to 0.10)	0.541
BSDS1 + AP1	0.23 (0.09 to 0.37)	0.001*	0.10 (−0.06 to 0.26)	0.216

*Note:* BSDS 1 = Non‐adherence to the Baltic Sea diet group (BSDS tertile 1). AP 1 = Advanced periodontitis group (at least one pocket depth ≥ 6 mm). Model 1: adjusted for energy intake. Adjusted *R* ^2^ = 0.005, *p*‐value < 0.001. Model 2: adjusted for baseline age, BMI, smoking status, physical activity, education level, CRP, HbA1c, medication of lipid‐modifying agents, and energy intake. Adjusted *R* ^2^ = 0.218, *p*‐value < 0.001.

Abbreviation: BSDS = Baltic Sea Diet Score.

* *p*‐value < 0.05.

**TABLE 3:** Associations of DII 3, advanced periodontitis, or combination of these both with serum CRP levels after 11 years of follow up.

Independent variable	Model 1		Model 2	
	Adjusted *β* (95% CI)	*p*	Adjusted *β* (95% CI)	*p*
DII 3	0.02 (−0.08 to 0.11)	0.762	−0.03 (−0.15 to 0.09)	0.629
AP 2	0.20 (0.09 to 0.31)	< 0.001*	−0.03 (−0.17 to 0.11)	0.655
DII3 + AP2	0.18 (0.02 to 0.34)	0.024*	0.01 (−0.17 to 0.19)	0.916

*Note:* DII 3 = Proinflammatory diet group (DII tertile 3). AP 2 = Advanced periodontitis group (at least one pocket depth ≥ 6 mm). Model 1: adjusted for energy intake. Adjusted *R* ^2^ = 0.004, *p*‐value = 0.002. Model 2: adjusted for baseline age, BMI, smoking status, physical activity, education level, CRP, HbA1c, medication of lipid‐modifying agents, and energy intake. Adjusted *R* ^2^ = 0.216, *p*‐value < 0.001.

Abbreviation: DII = Dietary Inflammatory index.

* *p*‐value < 0.05.

When the linear regression analysis was performed separately for men and women, the combination of BSDS 1 and AP 1 was statistically significantly associated with serum CRP in men (*β* = 0.23, *p*‐value = 0.011), but not in women (*β* = −0.01, *p*‐value = 0.994). The similar difference between genders was not observed when the combination of DII 3 and AP 2 and their association with serum CRP was analyzed in linear regression as the results remained statistically non‐significant both in men and women (*β* = 0.13, *p*‐value = 0.189 and *β* = −0.03, *p*‐value = 0.800, respectively).

## 4 Discussion

To our knowledge, the present study is the first to evaluate the cumulative effect of overall diet quality and periodontitis on serum CRP levels. The main finding was that advanced periodontitis, also when combined with non‐adherence to the Baltic Sea diet (assessed as BSDS 1) or proinflammatory diet (assessed as DII 3), was related to higher hs‐CRP levels at baseline. However, belonging to either the BSDS 1 or DII 3 group did not magnify the existing risk of developing elevated CRP levels in advanced periodontitis patients. Furthermore, when we analyzed the results from the 11‐year follow‐up after controlling for covariates, we failed to confirm significant associations between serum CRP levels and advanced periodontitis combined with indices of diet quality.

Studies evaluating associations of elevated inflammatory condition with concurrent exposure to overall diet quality and periodontitis are sparse. In a cross‐sectional study of 3238 adults from the NHANES 2009–2010 datasets, DII correlated with serum CRP concentrations, mean probing pocket depth, and mean clinical attachment loss (Machado et al. 2021). The authors also found that DII mediated the association between periodontal measurements and systemic inflammation levels. In the present study, however, the serum CRP levels were not higher in participants who were non‐adherent to the Baltic Sea diet or consumed the proinflammatory diet in comparison to the reference. Oxidative stress appears to play a contributing role in the pathogenesis of periodontal disease, causing damage in lipid‐, protein‐, and DNA‐components inside the cell and leading to compromised function of periodontal tissue (Shang, Liu, Zheng, and Zhang 2023). As diet quality may also increase or decrease the reactive oxygen species in tissues (Ilari et al. 2025), it was reasonable to assume that diet and periodontitis together would be associated with the marker of low‐grade systemic inflammation, reflected by CRP concentration.

The focus of BSDS is to evaluate the adherence to the Baltic Sea diet, while DII aims to determine the overall inflammatory potential of the diet. As the development of DII was based on the literature by evaluating each food component and their association with serum IL‐1β, IL‐4, IL‐6, IL‐10, TNF‐α, and CRP concentrations (Shivappa et al. 2014), it was expected that high DII scores would associate with higher serum CRP levels as well. Moreover, a cross‐sectional study using two independent cohorts of Finnish adults (*n* = 4579 and *n* = 1911) demonstrated an inverse association between BSDS and hs‐CRP concentrations (Kanerva, Loo, et al. 2014). However, the present study was unable to demonstrate an association between BSDS or DII and serum hs‐CRP. One possible explanation for the lack of association between diet quality and serum hs‐CRP levels could be the use of dietary tertiles. The first tertile of BSDS was used to indicate non‐adherence to BSDS while the third tertile of DII indicated a proinflammatory diet. The BSDS values in the first tertile ranged from 1 to 10 (out of 22), and therefore those participants may not be homogenous enough to accurately represent those with the unhealthiest diet. In addition, the DII scores in the third DII tertile ranged from −1.7 to 4.4. This means that some participants with the anti‐inflammatory diet (DII scores below zero) were also included in the third tertile. The use of tertiles, however, was chosen to facilitate interpretation and enable group comparisons. In addition, when the analyses were conducted by using the DII and BSDS as a continuous variables, the results remained similar.

Another possible explanation for the absence of a clear association between diet and CRP may be attributable to CRP's role as a nonspecific marker of inflammation. The CRP concentration increases in many chronic conditions, including autoimmune diseases, cardiovascular diseases, and inflammation (Rizo‐Téllez et al. 2023). In addition, psychosocial conditions other than diseases, including prolonged stress or frailty, affect serum CRP concentration (Johnson et al. 2013; Luo et al. 2024). Yet, serum CRP is widely used as an inflammatory biomarker in nutrition studies, elevated levels being associated with unhealthy dietary patterns (Hart et al. 2021a). However, including multiple inflammatory markers could have provided deeper insight into the association between diet and systemic inflammation, potentially revealing associations that were not detectable in the present study. A recent study analyzing 195 participants from the United Kingdom found no association between periodontitis and serum hs‐CRP, whereas a link was observed between periodontal status and systemic IL‐6 (Mainas et al. 2025). Similarly, no association was found between the adherence to the Mediterranean diet and assessed biomarkers in the same study, and these findings were consistent with those of the current research.

Even though DII has been associated with serum CRP in several large‐scale studies (Doustmohammadian et al. 2024; Lécuyer et al. 2023; Millar et al. 2022), there are controversial results as well. In a population‐based study of 2558 Australian adults, no association between DII and serum CRP levels was found (Hart et al. 2021b). The authors speculated that these results may be due to the complex nature of inflammation as well as that the overall effect of diet on CRP is small, and suggested accounting for various lifestyle factors being more crucial than focusing solely on diet when assessing inflammation. This perspective was addressed in the present study, but based on the results, we were not able to observe the increased serum CRP in those having two risk factors of inflammation when compared to only one risk factor.

According to our study, periodontitis at baseline was not associated with CRP after 11 years of follow‐up. This result was contractionary to a recent study of 3979 adults, showing baseline periodontitis to associate with higher CRP and a lesser decrease in CRP concentrations after 13 years of follow‐up (Molinsky et al. 2022). Moreover, substantial evidence supports an association between periodontitis and various systemic diseases, part of this relationship being mediated by inflammatory markers, including CRP (Hasan et al. 2025). Also, a recent follow‐up study of 4833 participants suggested serum CRP to be a potential biomarker to predict periodontitis development (Ghanem 2025). Finally, clinical designs of H2000 and H2011 surveys create a limitation. In both surveys, while periodontal pocket depths were measured at each tooth site, gingival inflammation was recorded per sextant, and clinical attachment levels, on the other hand, were not recorded (Suominen‐Taipale et al. 2008). The periodontal data available for the current analyses may hamper the interpretation of our results as well as their comparison with recent studies using other diagnostic criteria for periodontal disease.

In the present study, after controlling for potential confounding factors during the follow‐up period, the observed association between periodontitis and hs‐CRP was no longer statistically significant. In adjusted models, the main determinants of serum hs‐CRP were baseline BMI and hs‐CRP concentration. As the regression models included multiple covariates, there is a possibility of overadjustment (Schisterman et al. 2009). This can occur if the variable being adjusted for is actually part of the causal pathway. In such cases, the true association may be attenuated, as controlling for an intermediate variable can block part of the causal effect, potentially leading to a biased—or even null—estimate of the total effect. It is well known that a high BMI is strongly related to low‐grade systemic inflammation (Soták et al. 2024). It is also likely that BMI is related to diet and may act as one component in the causal pathway between diet and hs‐CRP. However, to examine this possibility more closely, the analyses were conducted also without adjusting with baseline BMI and the non‐significant results remained similar.

The diet quality was assessed by FFQ, which is prone to recall bias (Männistö et al. 1996). Also, as the study data were collected considerable time ago, it is possible that subsequent changes related to diet and lifestyle patterns have occurred, potentially limiting the generalizability of the findings to present‐day conditions. For example, dietary habits in Finland have improved over the past two decades, marked by a higher consumption of fruits and vegetables (Kaartinen et al. 2021 [In Finnish]). Nevertheless, current intakes do not meet the dietary guidelines, suggesting that many individuals still follow diets that may be inadequate—and potentially proinflammatory. Nonetheless, it cannot be ruled out that changes in diet over the 11‐year follow‐up period may have diluted the study results, particularly if participants' overall diet quality has improved. However, there is evidence that dietary habits are generally persistent and difficult to change (Stuber et al. 2023). Also, periodontitis remains a common oral disease (Suominen et al. 2025). Despite the extended period between the data collection and the present time, these findings offer insight into how the co‐occurrence of selected dietary indices and periodontitis is associated with serum hs‐CRP concentration. In addition, this study suggests some differences in the association between BSDS, periodontitis, and serum hs‐CRP in men and women, thereby offering some perspectives for future research.

The observational study design does not allow us to make any conclusions about causality. Even though the sampling of Health 2000 was carefully designed, and the sample was seen to represent well the target population (Aromaa 2004), the final sample included in the present study comprised only 3007 individuals. Gender distribution was similar in participants and those who were excluded, but the participants were younger (mean age 49 years compared to 58 years, *p* < 0.001), and they were more often having higher education (*p* < 0.001). This inevitably weakens the generalizability of the results, especially given that aging and lower education is associated with higher morbidity (Fonseca et al. 2020; Straka et al. 2024). Therefore, this study may underestimate the association between diet, periodontitis, and serum hs‐CRP, as those who are more likely to suffer chronic diseases or are at the highest risk to develop one, are missing. Despite these limitations, the strengths of our study include a large sample of the Finnish population, adjustment for relevant confounding factors, and an 11‐year follow‐up period, which gave us a longitudinal perspective as well.

It is well acknowledged that a healthy diet is beneficial for cardiovascular health and relates to a decreased risk of various chronic diseases (Shang, Liu, Zhu, et al. 2023; Tsao et al. 2023). Elevated plasma CRP levels have been linked to an increased prevalence of dyslipidemia, diabetes, and metabolic syndrome in Korean adults (Jeong et al. 2019), as well as to increased all‐cause mortality in the Danish population (Zacho et al. 2010). Within the limitations of the study, the baseline findings support the view that periodontitis should be considered as a systemic risk factor for chronic diseases, highlighting the importance of its early diagnosis and treatment. Nevertheless, the long‐term association between periodontitis, diet, and serum hs‐CRP could not be established, which might be due to limitations related to the definition of periodontitis or dietary categorization. The present study evaluated the serum hs‐CRP related to the diet and periodontitis. This is, however, only one perspective on the health effects of the diet. In future studies, the analysis of other “risk” components of the diet (e.g., high intake of saturated fats, low fiber intake, high sodium intake) concurrent with periodontitis could reveal synergistic effects of the diet and periodontitis on systemic health.

## 5 Conclusions

Concurrent exposure to two lifestyle‐related risk factors, namely non‐adherence to the Baltic Sea diet or adherence to a proinflammatory diet, and advanced periodontitis, was not clearly associated with increased serum hs‐CRP levels compared to the presence of only one risk factor. It is possible that their combined effect is too subtle to detect given the methodological limitations of the study.

## Author Contributions

Conceptualization: Sanna Syrjäläinen, Satu Männistö, Eija Könönen, and Ulvi Kahraman Gürsoy. Data acquisition: Satu Männistö and Anna Liisa Suominen. Formal analysis: Sanna Syrjäläinen. Project administration: Satu Männistö, Eija Könönen, and Ulvi Kahraman Gürsoy. Visualization: Sanna Syrjäläinen. Writing – original draft: Sanna Syrjäläinen. Writing – review and editing: Sanna Syrjäläinen, Satu Männistö, Eija Könönen, and Ulvi Kahraman Gürsoy. The final version of the manuscript was reviewed and approved by all authors.

## Funding

Funding for Health 2000 and Health 2011 studies was coordinated by the National Public Health Institute/KTL (currently the Finnish Institute for Health and Welfare/THL) through budgetary funds from the government and a grant from the Academy of Finland. Both studies also received funding from Finnish Centre for Pensions, the Local Government Pensions Institution, the UKK Institute, the Finnish Dental Association and the Finnish Dental Society, the Finnish Institute of Occupational Health, and the Finnish Work Environment Fund. Health 2000 was also funded by the Social Insurance Institution of Finland, the National Research and Development Centre for Welfare and Health, Statistics Finland, and the Occupational Safety and Health Fund of the State sector. Oral health examination in the Health 2000 Survey was partly funded by the Finnish Dental Association and the Finnish Dental Society Apollonia. Additionally, Health 2011 was funded by Minister of Social affairs and Health, and the Federation of Finnish Financial Services. S. Syrjäläinen received funding from the Minerva foundation (Finland), The Finnish Medical Foundation, and FINDOS‐Turku doctoral program (Finland). E. Könönen received funding form the Academy of Finland.

## Ethics Statement

The Health 2000 and Health 2011 surveys were approved by the Ethical Committee of the Hospital District of Helsinki and Uusimaa.

## Consent

Informed consent was obtained from all participants of the Health 2000 and Health 2011 surveys.

## Conflicts of Interest

The authors declare no conflicts of interest.

## Supporting information


**Table S1:** Regression coefficients from linear model with DII as predictor of serum C‐reactive protein by periodontitis severity.
**Table S2:** Regression coefficients from linear model with BSDS as predictor of serum C‐reactive protein by periodontitis severity.

## Data Availability

The data that support the findings of this study are available from the Finnish Institute for Health and Welfare (THL). Restrictions apply to the availability of these data, which were used under license for this study. Data can be requested at https://thl.fi/en/web/thlfi‐en/statistics‐and‐data/data‐and‐services with the permission of THL.

## References

[fsn371762-bib-0001] Aromaa, A. 2004. Health and Functional Capacity in Finland: Baseline Results of the Health 2000 Health Examination Survey. National Public Health Institute = Kansanterveyslaitos. https://www.julkari.fi/handle/10024/78534.

[fsn371762-bib-0002] Billingsley, H. E. , E. M. Heiston , M. P. Bellissimo , C. J. Lavie , and S. Carbone . 2024. “Nutritional Aspects to Cardiovascular Diseases and Type 2 Diabetes Mellitus.” Current Cardiology Reports 26, no. 3: 73–81. 10.1007/s11886-023-02018-x. 38261251 PMC10990987

[fsn371762-bib-0003] Black, S. , I. Kushner , and D. Samols . 2004. “C‐Reactive Protein*.” Journal of Biological Chemistry 279, no. 47: 48487–48490. 10.1074/jbc.R400025200. 15337754

[fsn371762-bib-0004] Blaum, C. , F. J. Brunner , F. Kröger , et al. 2021. “Modifiable Lifestyle Risk Factors and C‐Reactive Protein in Patients With Coronary Artery Disease: Implications for an Anti‐Inflammatory Treatment Target Population.” European Journal of Preventive Cardiology 28, no. 2: 152–158. 10.1177/2047487319885458. 33838040

[fsn371762-bib-0005] Calder, P. C. , R. Albers , J.‐M. Antoine , et al. 2009. “Inflammatory Disease Processes and Interactions With Nutrition.” British Journal of Nutrition 101, no. Suppl 1: S1–S45. 10.1017/S0007114509377867. 19586558

[fsn371762-bib-0006] Doustmohammadian, A. , B. Amirkalali , S. Esfandyari , et al. 2024. “The Association Between Dietary Inflammatory Index (DII) Scores and c‐Reactive Protein (CRP) and Nonalcoholic Fatty Liver Disease (NAFLD) in a General Population Cohort.” Clinical Nutrition ESPEN 60: 156–164. 10.1016/j.clnesp.2024.01.017. 38479904

[fsn371762-bib-0007] Drozd, M. , M. Pujades‐Rodriguez , A. W. Morgan , et al. 2022. “Systemic Inflammation Is Associated With Future Risk of Fatal Infection: An Observational Cohort Study.” Journal of Infectious Diseases 226, no. 3: 554–562. 10.1093/infdis/jiac186. 35535512 PMC9417123

[fsn371762-bib-0008] Fonseca, R. , P.‐C. Michaud , and Y. Zheng . 2020. “The Effect of Education on Health: Evidence From National Compulsory Schooling Reforms.” SERIEs 11, no. 1: 83–103. 10.1007/s13209-019-0201-0.

[fsn371762-bib-0009] Forouhi, N. G. 2023. “Embracing Complexity: Making Sense of Diet, Nutrition, Obesity and Type 2 Diabetes.” Diabetologia 66, no. 5: 786–799. 10.1007/s00125-023-05873-z. 36786838 PMC9925928

[fsn371762-bib-0010] Furman, D. , J. Campisi , E. Verdin , et al. 2019. “Chronic Inflammation in the Etiology of Disease Across the Life Span.” Nature Medicine 25: 12. 10.1038/s41591-019-0675-0. PMC7147972 31806905

[fsn371762-bib-0011] Ghanem, A. S. 2025. “The Role of Systemic Health Indicators, Including C‐Reactive Protein and eGFR, in Predicting Periodontal Disease: A Longitudinal Study.” International Journal of Molecular Sciences 26, no. 2: 741. 10.3390/ijms26020741. 39859455 PMC11766259

[fsn371762-bib-0012] Hajishengallis, G. 2022. “Interconnection of Periodontal Disease and Comorbidities: Evidence, Mechanisms, and Implications.” Periodontology 2000 89, no. 1: 9–18. 10.1111/prd.12430. 35244969 PMC9018559

[fsn371762-bib-0056] Haro, A. , T. Saxlin , A. Suominen , et al. 2012. “Serum Lipids Modify Periodontal Infection – C‐Reactive Protein Association.” Journal of Clinical Periodontology 39, no. 9: 817–823. 10.1111/j.1600-051x.2012.01920.x. 22780440

[fsn371762-bib-0013] Hart, M. J. , S. J. Torres , S. A. McNaughton , and C. M. Milte . 2021a. “A Dietary Inflammatory Index and Associations With C‐Reactive Protein in a General Adult Population.” European Journal of Nutrition 60, no. 7: 4093–4106. 10.1007/s00394-021-02573-5. 33991227

[fsn371762-bib-0014] Hart, M. J. , S. J. Torres , S. A. McNaughton , and C. M. Milte . 2021b. “Dietary Patterns and Associations With Biomarkers of Inflammation in Adults: A Systematic Review of Observational Studies.” Nutrition Journal 20, no. 1: 24. 10.1186/s12937-021-00674-9. 33712009 PMC7955619

[fsn371762-bib-0015] Hasan, F. , A. Tandon , H. AlQallaf , V. John , M. Sinha , and M. P. Gibson . 2025. “Inflammatory Association Between Periodontal Disease and Systemic Health.” Inflammation 48: 3763–3775. 10.1007/s10753-025-02317-1. 40768114 PMC12722499

[fsn371762-bib-0016] Heistaro, S. 2008. “Methodology Report: Health 2000 Survey.” https://www.julkari.fi/handle/10024/78185.

[fsn371762-bib-0017] Ilari, S. , S. Proietti , F. Milani , et al. 2025. “Dietary Patterns, Oxidative Stress, and Early Inflammation: A Systematic Review and Meta‐Analysis Comparing Mediterranean, Vegan, and Vegetarian Diets.” Nutrients 17, no. 3: 548. 10.3390/nu17030548. 39940408 PMC11819869

[fsn371762-bib-0055] Jaedicke, K. M. , P. M. Preshaw , and J. J. Taylor . 2015. “Salivary Cytokines as Biomarkers of Periodontal Diseases.” Periodontology 2000 70, no. 1: 164–183. 10.1111/prd.12117. 26662489

[fsn371762-bib-0018] Jeong, H. , S.‐Y. Baek , S. W. Kim , et al. 2019. “C Reactive Protein Level as a Marker for Dyslipidaemia, Diabetes and Metabolic Syndrome: Results From the Korea National Health and Nutrition Examination Survey.” BMJ Open 9, no. 8: e029861. 10.1136/bmjopen-2019-029861. PMC6720331 31473619

[fsn371762-bib-0019] Johnson, T. V. , A. Abbasi , and V. A. Master . 2013. “Systematic Review of the Evidence of a Relationship Between Chronic Psychosocial Stress and C‐Reactive Protein.” Molecular Diagnosis & Therapy 17, no. 3: 147–164. 10.1007/s40291-013-0026-7. 23615944

[fsn371762-bib-0020] Kaartinen, N. , S. Männistö , H. Reinivuo , et al. 2021. “Aikuisväestön Ruoankäytön Ja Ravintoaineiden Saannin Muutokset Vuosina 1997–2017: Kansallinen FinRavinto‐Tutkimus.” Lääkärilehti 5, no. 76: 273–280.

[fsn371762-bib-0021] Kaartinen, N. E. , H. Tapanainen , L. M. Valsta , et al. 2012. “Relative Validity of a FFQ in Measuring Carbohydrate Fractions, Dietary Glycaemic Index and Load: Exploring the Effects of Subject Characteristics.” British Journal of Nutrition 107, no. 9: 1367–1375. 10.1017/S0007114511004296. 21899807

[fsn371762-bib-0022] Kanerva, N. , N. E. Kaartinen , U. Schwab , M. Lahti‐Koski , and S. Männistö . 2014. “The Baltic Sea Diet Score: A Tool for Assessing Healthy Eating in Nordic Countries.” Public Health Nutrition 17, no. 8: 1697–1705. 10.1017/S1368980013002395. 24172174 PMC10282237

[fsn371762-bib-0023] Kanerva, N. , B.‐M. Loo , J. G. Eriksson , et al. 2014. “Associations of the Baltic Sea Diet With Obesity‐Related Markers of Inflammation.” Annals of Medicine 46, no. 2: 90–96. 10.3109/07853890.2013.870020. 24447090

[fsn371762-bib-0054] Könönen, E. , M. Gursoy , and U. Gursoy . 2019. “Periodontitis: A Multifaceted Disease of Tooth‐Supporting Tissues.” Journal of Clinical Medicine 8, no. 8: 1135. 10.3390/jcm8081135. 31370168 PMC6723779

[fsn371762-bib-0024] Lécuyer, L. , N. Laouali , V. Viallon , et al. 2023. “Associations Between Dietary Inflammatory Scores and Biomarkers of Inflammation in the European Prospective Investigation Into Cancer and Nutrition (EPIC) Cohort.” Clinical Nutrition (Edinburgh, Scotland) 42, no. 7: 1115–1125. 10.1016/j.clnu.2023.05.012. 37271707

[fsn371762-bib-0025] Lundqvist, A. , and T. Mäki‐Opas . 2016. “Health 2011 Survey—Methods [D6].” THL. https://www.julkari.fi/handle/10024/130780.

[fsn371762-bib-0026] Luo, Y.‐F. , Z.‐J. Cheng , Y.‐F. Wang , et al. 2024. “Unraveling the Relationship Between High‐Sensitivity C‐Reactive Protein and Frailty: Evidence From Longitudinal Cohort Study and Genetic Analysis.” BMC Geriatrics 24, no. 1: 222. 10.1186/s12877-024-04836-2. 38439017 PMC10913347

[fsn371762-bib-0027] Machado, V. , J. Botelho , J. Viana , et al. 2021. “Association Between Dietary Inflammatory Index and Periodontitis: A Cross‐Sectional and Mediation Analysis.” Nutrients 13, no. 4: 1194. 10.3390/nu13041194. 33916342 PMC8066166

[fsn371762-bib-0028] Mainas, G. , G. Grosso , J. Di Giorgio , et al. 2025. “Relationship Between Mediterranean Diet and Periodontal Inflammation in a UK Population: A Cross‐Sectional Study.” Journal of Periodontology 97: 85. 10.1002/jper.70016. 40952033 PMC12902710

[fsn371762-bib-0029] Männistö, S. , K. Harald , T. Härkänen , et al. 2021. “Association Between Overall Diet Quality and Postmenopausal Breast Cancer Risk in Five Finnish Cohort Studies.” Scientific Reports 11: 16718. 10.1038/s41598-021-95773-2. 34408173 PMC8373908

[fsn371762-bib-0030] Männistö, S. , M. Virtanen , T. Mikkonen , and P. Pietinen . 1996. “Reproducibility and Validity of a Food Frequency Questionnaire in a Case‐Control Study on Breast Cancer.” Journal of Clinical Epidemiology 49, no. 4: 401–409. 10.1016/0895-4356(95)00551-x. 8621990

[fsn371762-bib-0031] Millar, S. R. , P. Navarro , J. M. Harrington , et al. 2022. “Dietary Score Associations With Markers of Chronic Low‐Grade Inflammation: A Cross‐Sectional Comparative Analysis of a Middle‐ to Older‐Aged Population.” European Journal of Nutrition 61, no. 7: 3377–3390. 10.1007/s00394-022-02892-1. 35511284 PMC9464136

[fsn371762-bib-0032] Minihane, A. M. , S. Vinoy , W. R. Russell , et al. 2015. “Low‐Grade Inflammation, Diet Composition and Health: Current Research Evidence and Its Translation.” British Journal of Nutrition 114, no. 7: 999–1012. 10.1017/S0007114515002093. 26228057 PMC4579563

[fsn371762-bib-0033] Molinsky, R. L. , M. Yuzefpolskaya , F. L. Norby , et al. 2022. “Periodontal Status, C‐Reactive Protein, NT‐proBNP, and Incident Heart Failure: The ARIC Study.” JACC Heart Failure 10, no. 10: 731–741. 10.1016/j.jchf.2022.05.008. 36175058 PMC9976480

[fsn371762-bib-0034] National Nutrition Council . 2021. “Finnish Food Authority.” https://www.ruokavirasto.fi/en/foodstuffs/healthy‐diet/national‐nutrition‐council/.

[fsn371762-bib-0036] Reinivuo, H. , T. Hirvonen , M.‐L. Ovaskainen , T. Korhonen , and L. M. Valsta . 2010. “Dietary Survey Methodology of FINDIET 2007 With a Risk Assessment Perspective.” Public Health Nutrition 13, no. 6A: 915–919. 10.1017/S1368980010001096. 20513260

[fsn371762-bib-0037] Ridker, P. M. 2016. “A Test in Context: High‐Sensitivity C‐Reactive Protein.” Journal of the American College of Cardiology 67, no. 6: 712–723. 10.1016/j.jacc.2015.11.037. 26868696

[fsn371762-bib-0038] Rizo‐Téllez, S. A. , M. Sekheri , and J. G. Filep . 2023. “C‐Reactive Protein: A Target for Therapy to Reduce Inflammation.” Frontiers in Immunology 14: 1237729. 10.3389/fimmu.2023.1237729. 37564640 PMC10410079

[fsn371762-bib-0039] Salzberg, T. N. , B. T. Overstreet , J. D. Rogers , J. V. Califano , A. M. Best , and H. A. Schenkein . 2006. “C‐Reactive Protein Levels in Patients With Aggressive Periodontitis.” Journal of Periodontology 77, no. 6: 933–939. 10.1902/jop.2006.050165. 16734565

[fsn371762-bib-0040] Schisterman, E. F. , S. R. Cole , and R. W. Platt . 2009. “Overadjustment Bias and Unnecessary Adjustment in Epidemiologic Studies.” Epidemiology 20, no. 4: 488–495. 10.1097/EDE.0b013e3181a819a1. 19525685 PMC2744485

[fsn371762-bib-0041] Seth, C. , V. Schmid , S. Mueller , et al. 2025. “Diabetes, Obesity, and Cardiovascular Disease—What Is the Impact of Lifestyle Modification?” Herz 50, no. 4: 240–245. 10.1007/s00059-025-05309-x. 40085207

[fsn371762-bib-0042] Shang, J. , H. Liu , Y. Zheng , and Z. Zhang . 2023. “Role of Oxidative Stress in the Relationship Between Periodontitis and Systemic Diseases.” Frontiers in Physiology 14: 1210449. 10.3389/fphys.2023.1210449. 37501927 PMC10369007

[fsn371762-bib-0043] Shang, X. , J. Liu , Z. Zhu , et al. 2023. “Healthy Dietary Patterns and the Risk of Individual Chronic Diseases in Community‐Dwelling Adults.” Nature Communications 14: 6704. 10.1038/s41467-023-42523-9. PMC10593819 37872218

[fsn371762-bib-0044] Shivappa, N. , S. E. Steck , T. G. Hurley , J. R. Hussey , and J. R. Hébert . 2014. “Designing and Developing a Literature‐Derived, Population‐Based Dietary Inflammatory Index.” Public Health Nutrition 17, no. 8: 1689–1696. 10.1017/S1368980013002115. 23941862 PMC3925198

[fsn371762-bib-0045] Soták, M. , M. Clark , B. E. Suur , and E. Börgeson . 2024. “Inflammation and Resolution in Obesity.” Nature Reviews Endocrinology 21: 45. 10.1038/s41574-024-01047-y. 39448830

[fsn371762-bib-0046] Straka, J. , L. Šídlo , and I. Kulhánová . 2024. “Trends in Healthy Life Years Between 2005 and 2019 in 31 European Countries: The Compression or Expansion of Morbidity?” International Journal of Public Health 69: 1607574. 10.3389/ijph.2024.1607574. 39479338 PMC11521812

[fsn371762-bib-0047] Stuber, J. M. , J. Lakerveld , J. W. Beulens , and J. D. Mackenbach . 2023. “Better Understanding Determinants of Dietary Guideline Adherence Among Dutch Adults With Varying Socio‐Economic Backgrounds Through a Mixed‐Methods Exploration.” Public Health Nutrition 26, no. 6: 1172–1184. 10.1017/S1368980023000228. 36700250 PMC10348427

[fsn371762-bib-0048] Suominen, A. L. , A. Leskinen , T. Saxlin , et al. 2025. “Dental and Periodontal Condition by Sociodemographics in Finnish Adults in 2023: Cross‐Sectional Results From the Healthy Finland Survey.” Acta Odontologica Scandinavica 84: 457–470. 10.2340/aos.v84.44370. 40827429 PMC13063821

[fsn371762-bib-0049] Suominen‐Taipale, L. , A. Nordblad , M. Vehkalahti , and A. Aromaa . 2008. Oral Health in the Finnish Adult Population: Health 2000 Survey [C2_Toimitettu Kirja, Kokoomateos, Konferenssijulkaisu Tai Lehden Erikoisnumero (Book (Editor))]. Kansanterveyslaitos. https://www.julkari.fi/handle/10024/103030.

[fsn371762-bib-0050] Syrjäläinen, S. , S. Männistö , E. Könönen , et al. 2024. “Dietary Inflammatory Index in Relation to Salivary Cytokine Concentrations and Periodontitis: A Cross‐Sectional Analysis.” Journal of Clinical Periodontology 51, no. 4: 406–416. 10.1111/jcpe.13917. 38158626

[fsn371762-bib-0051] Tsao, C. W. , A. W. Aday , Z. I. Almarzooq , et al. 2023. “Heart Disease and Stroke Statistics‐2023 Update: A Report From the American Heart Association.” Circulation 147, no. 8: e93–e621. 10.1161/CIR.0000000000001123. 36695182 PMC12135016

[fsn371762-bib-0052] Zacho, J. , A. Tybjærg‐Hansen , and B. G. Nordestgaard . 2010. “C‐Reactive Protein and All‐Cause Mortality—The Copenhagen City Heart Study.” European Heart Journal 31, no. 13: 1624–1632. 10.1093/eurheartj/ehq103. 20423919

[fsn371762-bib-0053] Zhu, G. , K. Yang , T. Liu , et al. 2025. “Causal Network Between Periodontitis and Systemic Inflammation: Triangulating Evidence From Mendelian Randomization and Sequencing Datasets.” Journal of Periodontology 96: 1432–1444. 10.1002/JPER.24-0382. 40627758

