# Supplementary material for: Diet Quality and Advanced Periodontitis in Relation to Serum CRP Levels: An 11‐Year Follow‐Up Study
Source: Food Sci Nutr. 2026 Apr 13;14(4):e71762. doi: 10.1002/fsn3.71762 (PMC13071855; doi:10.1002/fsn3.71762)
Supplement: Supplementary file 1 — Table S1: Regression coefficients from linear model with DII as predictor of serum C‐reactive protein by periodontitis severity. Table S2: Regression coefficients from linear model with BSDS as predictor of serum C‐reactive protein by periodontitis severity. [file FSN3-14-e71762-s001.docx]

Supporting information

Table S1. Regression coefficients from linear model with DII as predictor of serum C-reactive protein by periodontitis severity

|  | Model 1 |  | Adj.  R^2^ | Model 2 |  | Adj. R^2^ |
| --- | --- | --- | --- | --- | --- | --- |
| Periodontitis severity | Adjusted β  (95 % CI) | P-value |  | Adjusted β  (95 % CI) | P-value |  |
| Non periodontitis | -0.01 | 0.720 | -0.00 | 0.002 | 0.915 | 0.23 |
| Moderate periodontitis | -0.01 | 0.613 | -0.00 | -0.005 | 0.775 | 0.22 |
| Advanced periodontitis | 0.02 | 0.524 | -0.003 | 0.01 | 0.695 | 0.11 |

*Adj. R^2^= Adjusted R^2^, DII=Dietary inflammatory index, CI= Confidence interval*

*Non-periodontitis= Probing pocket depths (PPD) ≤ 3 mm at all teeth, moderate periodontitis = PPD of 4-5 mm at any tooth, advanced periodontitis = PPD ≥ 6 mm at any tooth*

*Model 1: adjusted for energy intake.*

*Model 2: adjusted for baseline age, BMI, smoking status, physical activity, education level, CRP, HbA1c, medication of lipid-modifying agents, and energy intake*

Table S2. Regression coefficients from linear model with BSDS as predictor of serum C-reactive protein by periodontitis severity

|  | Model 1 | | | Model 2 | | |
| --- | --- | --- | --- | --- | --- | --- |
| Periodontitis severity | Adjusted β  (95 % CI) | P-value | Adj.  R^2^ | Adjusted β  (95 % CI) | P-value | Adj. R^2^ |
| Non periodontitis | -0.01 | 0.507 | 0.00 | -0.010 | 0.247 | 0.23 |
| Moderate periodontitis | -0.00 | 0.847 | -0.00 | -0.01 | 0.409 | 0.22 |
| Adcanced periodontitis | 0.00 | 0.937 | -0.00 | 0.00 | 0.760 | 0.11 |

*Adj. R^2^= Adjusted R^2^, BSDS = Baltic Sea Diet Score, CI= Confidence interval*

*Non-periodontitis= Probing pocket depths (PPD) ≤ 3 mm at all teeth, moderate periodontitis = PPD of 4-5 mm at any tooth, advanced periodontitis = PPD ≥ 6 mm at any tooth*

*Model 1: adjusted for energy intake.*

*Model 2: adjusted for baseline age, BMI, smoking status, physical activity, education level, CRP, HbA1c, medication of lipid-modifying agents, and energy intake*
